# The socio-technical organisation of community pharmacies as a factor in the Electronic Prescription Service Release Two implementation: a qualitative study

**DOI:** 10.1186/1472-6963-12-471

**Published:** 2012-12-20

**Authors:** Jasmine Harvey, Anthony J Avery, Justin Waring, Nick Barber

**Affiliations:** 1 Department of Social Science, School of Social Geographical and Political Sciences, Loughborough University, 3rd Floor, Brockington Building, Loughborough LE11 3TU, UK; 2 School of Community Health Sciences, Division of Primary Care, University of Nottingham, Queens Medical Centre, Nottingham NG7 2UH, UK; 3 Nottingham University Business School, Jubilee Campus, Nottingham, NG8 1BB, UK; 4 Department of Practice and Policy, UCL School of Pharmacy, Mezzanine Floor, BMA House, Tavistock Square, London, WC1H 9JP, UK

**Keywords:** Community pharmacy, EPS2, Socio-technical, Work practice

## Abstract

**Background:**

The introduction of a new method of transmitting prescriptions from general practices to community pharmacies in England (Electronic Prescription Service Release 2 (EPS2)) has generated debate on how it will change work practice. As EPS2 will be a key technical element in dispensing, we reviewed the literature to find that there were no studies on how social and technical elements come together to form work practice in community pharmacies. This means the debate has little point of reference. Our aim therefore was to study the ways social and technical elements of a community pharmacy are used to achieve dispensing through the development of a conceptual model on pharmacy work practice, and to consider how a core technical element such the EPS2 could change work practice.

**Method:**

We used ethnographic methods inclusive of case-study observations and interviews to collect qualitative data from 15 community pharmacies that were in the process of adopting or were soon to adopt EPS2. We analysed the case studies thematically and used rigorous multi-dimensional and multi-disciplinary interpretive validation techniques to cross analyse findings.

**Results:**

In practice, dispensing procedures were not designed to take into account variations in human and technical integration, and assumed that repetitive and collective use of socio-technical elements were at a constant. Variables such as availability of social and technical resources, and technical know-how of staff were not taken into account in formalised procedures. Yet community pharmacies were found to adapt their dispensing in relation to the balance of social and technical elements available, and how much of the social and technical elements they were willing to integrate into dispensing. While some integrated as few technical elements as possible, some depended entirely on technical artefacts. This pattern also applied to the social elements of dispensing. Through the conceptual model development process, we identified three approaches community pharmacies used to appropriate procedures in practice. These were ‘technically oriented’, ‘improvising’ or ‘socially oriented’.

**Conclusion:**

We offer a model of different work approaches community pharmacies use to dispense, which suggests that when adopting a core technical element such as the EPS2 system of dispensing there could be variations in its successful adoption. Technically oriented pharmacies might find it easiest to integrate a similar artefact into work practice although needs EPS2 to synchronise effectively with existing technologies. Pharmacies adopting an improvising-approach have the potential to improve how they organise dispensing through EPS2 although they will need to improve how they apply their operating procedures. Socially oriented pharmacies will need to dramatically adapt their approach to dispensing since they usually rely on few technical tools.

## Background

Community pharmacies in England are currently undergoing a profound change through the introduction of a system for receiving prescriptions electronically from general practices (the Electronic Prescription Service Release 2 (EPS2) implementation). The EPS2 is an automated system that enables prescribers to digitally sign and send prescriptions to a central database called the Spine. Prescriptions on the Spine can be downloaded and dispensed by a pharmacy – nominated by the patient [1-3]. Whilst there are various debates on how the EPS2 could change pharmacy work practice both positively or negatively, there is little data in the pharmacy context to inform these debates. For example official channels such as Connecting for Health tend to promote EPS2 benefits [4], while discussions in pharmacy forums have projected dystopian visions of the EPS2 [5]. Studies of similar systems in healthcare settings show that successful implementation is not down to user perception alone, but comprises other key variables such as management processes and internal structures [6-9].

The EPS2 is a core technical element that will be adapted into practice and yet a review of the literature shows few previous studies on how social and technical elements come together in community pharmacies. In addition, it is generally assumed in the pharmacy practice literature that Standard Operating Procedures (SOPs) used in pharmacies to safely dispense medications are strictly followed, meaning the EPS2 will be expected to slot into these sets of procedures. Yet studies have shown that people usually adapt their work procedures and protocols to suit them [10-12]. Brown and Duguid for example contend that formal descriptions of work (procedures or protocols) tended to be abstracted from actual practice, thereby removing the unique practices or intricacies through which work is achieved within an environment [12].

In this study, we took a socio-technical approach to qualitatively study how people, technical systems and linked dispensing procedures in community pharmacies come together to form work practice. From this, we have developed a conceptual model, taking into account repetitive and collective use of socio-technical resources and processes, in relation to organisational behaviour towards dispensing [13,14]. The second part of our study addressed how factors such as the socio-technical organisation of a community pharmacy could influence the successful adoption of EPS2 in practice. Please note that while we took a socio-technical approach, there are instances in the paper where we have had to differentiate between the ‘social’ and the ‘technical’ as separate entities.

## Methods

More than 300 hours were spent by the research team collecting qualitative data in the form of ethnographical observations and interviews. Data collection was organised as site visits lasting two-three days with each of the 15 community pharmacies. At the end of each visit, an audio recorded interview (average 10 minutes), with at least one pharmacy professional, was conducted to verify field notes and explore their knowledge of EPS2. For example, their knowledge of how EPS2 may benefit dispensing and any concerns they have. The 15 sites studied were purposively sampled according to size, geographic location and ownership (Table 1). The study protocol was submitted to an NHS Research Ethics Committee and was classed as a service evaluation. NHS Research and Development approvals were obtained prior to conducting data collection of the various study sites. The research was designed by sociologists, patient safety and pharmacy practice experts, and the primary researcher (data collector) was a social scientist specifically trained in using ethnological methods to study socio-technological processes in communities of practice. The observations used unobtrusive qualitative data collection methods, chiefly non-participant observation and shadowing [15-17], and were written up as case studies following particular themes:

• Physicality of the pharmacy – this consisted of geographic location, approximate size in square meters, ownership of the pharmacy, a representative drawing of the floor layout and organisation of the space.

• Workflow – fluidity of the dispensing processes from when the prescription arrived in the pharmacy to when it was dispensed for both acute and repeat prescriptions. Walk-in (usually acute) prescription dispensing was timed as dispensing journeys.

• Workload - the types and amount of dispensing conducted, hours of work, and approximate items dispensed monthly.

• Resources – the number of professionals present at the time of observation ranging from the counter assistant to the responsible pharmacist including delivery drivers, and other resources such as printers, computers, controlled drugs fridge, account books and pharmacy management software.

• Engagement with electronic aspects of dispensing – how CP professionals used the technical elements to aid the dispensing process. Technical systems included any electronic tools and coloured basket systems.

• Social elements of dispensing – how CP professionals interacted with each other, and patients/customers during dispensing were recorded in the temporal context.

**Table 1:** Data collection sites

**Case Study**	**Location**	**Number of staff**	**Number of dispensing staff (including pharmacist)**	**Assigned Delivery driver(s)**	**Ownership**^**a**^
**1**	Town–Suburban	2–5	2–4	Yes	Independent
**2**	Town-Health Centre	5–10	5–7	Yes	Independent
**3**	Town–High street	2–4	2–4	Yes	Chain
**4**	City–Suburban	2–5	2–4	Yes	Chain
**5**	Town–High street	5–10	5–6	Yes	Chain
**6**	City–Inner city	2–5	2–4	No	Independent
**7**	Town–Suburban	2–4	2–3	No	Independent
**8**	Town-Shopping Centre	2–3	2–3	No	Chain
**9**	City–Residential	2–4	2–4	Yes	Chain
**10**	Village–High street	2–4	2–4	Yes	Independent
**11**	City–Residential	4–6	4–6	Yes	Chain
**12**	Village–centre	2–4	2–4	Yes	Chain
**13**	City –Inner city	2–3	2–3	Yes	Local chain
**14**	City Inner city	2–3	2–3	Yes	Local chain
**15**	City - Suburban	4–5	2–3	No	Independent

^a^Ownership is used to denote the pharmacy’s status. Some pharmacies were owned by individuals and these are referred to as ‘independent’. Others were owned by chain organisations and often consisted of multiple pharmacies under the same organisation across communities and geographical areas. These are referred to as ‘chain’.

These themes were chosen as a result of a comprehensive review of the socio-technical literature such as Mackenzie and Wajcman [11] and May et al. [18]; in combination with review of the international literature on the various aspects on pharmacy practice [19-26]. The comprehensive review showed that although dispensing had been researched in various contexts, there was no literature on how linked processes in dispensing formed work practice taking into account the socio-technical organisation of the pharmacy. This included how people, the technical systems and the processes come together to form work practice hence we concentrated on these elements in our study. Additional file 1: Appendix 1 shows a short section of field notes to illustrate the data collection format (please note that it has been edited to remove all identifiable, and commercially sensitive information).

A textual analysis was conducted using manual coding on the themes in each case study, and interpretively validated using multi-dimensional and multi-disciplinary cross-case analysis that included:

– Experts - practising pharmacists, academic pharmacist, pharmacy technicians and medication safety professionals;

– Interviews conducted during the observation period; and

– Multi-disciplinary data verification from social science, information systems and EPS2 fields.

To develop the conceptual model, analysis was firstly conducted on the repetitive socio-technical elements in dispensing. We used data on pharmacy characteristics to create social and technical diagrams that showed which ‘people’ elements and technologies were used to achieve dispensing tasks. Secondly we looked at how dispensing was achieved through collective use of the social and technical elements identified in the diagrams, to ascertain socio-technical interdependency. Thirdly we mapped each pharmacy’s socio-technical characteristic onto a socio-technical spectrum to develop the model of pharmacy approaches to work.

## Results and discussion

In the following four sections, we describe and discuss our analyses and results. The first section addresses how descriptive social and technical characteristics were *repetitively* used to achieve dispensing functions. The second section describes how social and technical characteristics *collectively* came together in themes that were studied, using comprehensive headings. The third section shows how the repetitive and collective characteristics were used to create pharmacy work approaches model using a spectrum. The fourth section describes how a technical artefact such as the EPS2 could be adapted into each work approach if EPS2 is rolled out to all community pharmacies in the future.

### Repetitive requirements: Social and technical frameworks in dispensing

Using the pharmacies’ descriptive characteristics, we analysed the social and technical elements that typified dispensing through their repetitive or recurring use in dispensing processes. This included types of technical tools used, technical support, software, hardware, people involved, staff, support staff and space. The purpose of this was to establish how social and technical elements were *repetitively* used to deliver the same functions or tasks in dispensing. Our ideas on what constitutes social structure and technical systems of work were influenced by pioneers of socio-technical studies such as Wajcman and Mackenzie [11] Pinch and Bijker [27] and Kling [28]. In these literatures, a social group was generally described as organisation of humans or relevant groups or members of a community that connect and support that community. A technology was described as consisting of a physical artefact, processes involving the artefact and knowledge on operating the artefact.

In our analysis of the elements that were repetitively used to complete dispensing functions, we firstly looked at ‘people’ or social resources and what processes and elements (both tangible and intangible) they used achieve tasks. The people that recurred in our observations were pharmacists, pharmacy proprietors, customers, medicines counter assistants, dispensers, repeat prescription collection and delivery drivers, and drug delivery drivers. On two occasions relatives visited staff but this was excluded from the analysis as it was not common across the case studies. The people element interacted at various levels between themselves and the physical artefacts to achieve their work.

We then looked at all the technical (electronic and physical) elements that were repeatedly required to achieve dispensing tasks. From our recordings, the ones that recurred were information and communication media such as telephones and fax, pharmacy manager software, the physical computers, physical storage equipment such as controlled drugs fridge, medicines storage units (including a robot), and, CCTV cameras used to monitor the immediate environment. Staff at one pharmacy listened to the radio, and at another watched TV during dispensing, but these were not repetitively used across the case studies and were therefore excluded from the analysis. The findings showed how both social and technical elements interdependently achieve the same dispensing functions.

**Figure 1:**
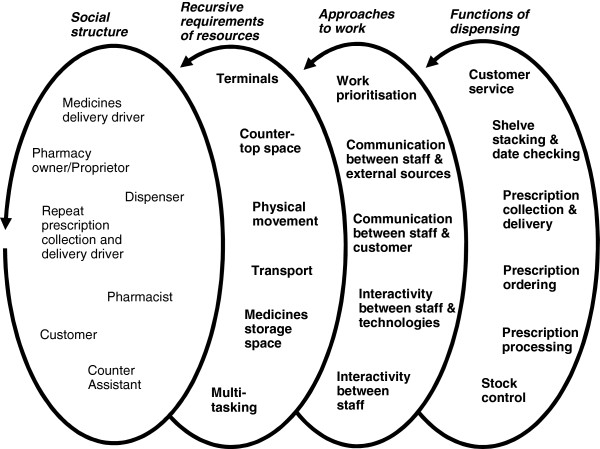
People-centred framework showing social dynamics observed in pharmacy work practice.

In the social element, people characterise the approach to work. This included people that were part of dispensing such as customers, delivery drivers, counter assistants, dispensers, pharmacists and pharmacy proprietors. Dispensing functions were achieved through repetitive resources required by the staff. This included how they needed each other, space, and technical artefacts. The approach to work was centred on interaction and communication among various people to perform functions of work, in this case dispensing. Figure 1 shows how the ‘people’ element was used to achieve work.

**Figure 2:**
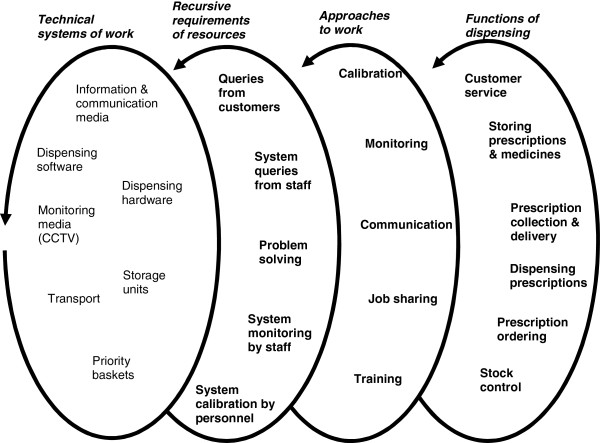
Techno-centred framework showing technical processes observed in pharmacy work practice.

The technical framework comprised of the technical systems through which work was carried out by the staff, the customer and other people. Technologies of work included both technical software and hardware of dispensing, and other technical systems of work such as prioritisation of work using baskets and storage. Similar to the social framework, the technical framework was integral to how work was characterised and approached through technical elements. Figure 2 show how technical elements repetitively relied on resources such as querying, calibrating, and monitoring the systems.

In Figures 1 and 2,

• The two left-hand side loops of both diagrams show the socio-technical interdependence within the dispensing environment such as social elements requiring some technical elements and vice versa;

• The diagrams then show through ‘approaches to work’, how this interdependence could a) use as little of the technical elements as possible, thereby mostly relying on effective communication between the human elements, such as frequent consultations to complete tasks (Figure 1); or b) incorporate as many technical elements as possible, thereby mostly relying on effective human-technological interactivity. For example, a calibrated colour-basket system communicating that there are exactly five prescriptions left to dispense, and that three are repeat prescriptions for a surgery because they are in white baskets, and two are urgent and need immediate dispensing because they are in red baskets (Figure 2);

• The diagrams then shows ways both approaches are used to achieve the same dispensing tasks.

The diagrams demonstrate how interlinked groups of resources and processes might be required to complete dispensing tasks using open-ended loops to suggest the existence of other factors.

### Collective use: social and technical elements in dispensing

We analysed how the repetitive elements identified were collectively used in each theme. For example, how did the social and technical elements come together in the ‘workload’ theme in all 15 case studies? The purpose of this was to identify how each data collection theme reflected socio-technical interdependency. For instance, managing a pharmacy workload needs both people and technical elements but within this theme, advising a patient in store may require less use of a technical element than when dispensing bulk prescriptions. The focus, therefore, was on how socio-technical elements were *collectively* used to achieve dispensing tasks.

The study showed that the way in which socio-technical elements were collectively used in tasks was important to how dispensing was practiced. Various amounts of both elements were adapted into work depending on which elements were available to the pharmacy, which elements people wanted to adapt into work, and how much of that element they wanted to adapt into work. In the analysis, new and interlinked themes emerged. For example, while we recorded resources such as staff and instruments, we also recorded how the work was distributed. This excerpt from Case 05 demonstrates this: “10.00, there are two dispensers at the back room. One is re-stocking shelves with medicines and the other is processing medicines. There are 14 red baskets on the back counter of the back room near processing terminal. The baskets are being processed by the dispenser”. We also found aspects of the physicality of the pharmacy linked to this. For example, sometimes it was impossible for two dispensers to move around at the same time due to the small physical space; work was therefore distributed with space taken into account. The comprehensive heading ‘*resource and distribution of work’* brings all these collective use of socio-technical resources under one heading. Similarly, in the workflow and workload themes, how work was *prioritised and organised* using both social and technical elements to form protocols was the common theme. The part people played in the social engagement theme, and how they adapted technical elements to suit them such as job sharing multi-tasking, are grouped into *Occupational tasks.* How technologies were engaged with by the people to complete tasks are grouped into *engagement with technical systems.* Each of these themes is discussed further below.

In resource and distribution of work, a key resource was how the physical space was used: space for physical movement, storage and processing medicines aided the fluidity of the dispensing process. In cases where all three spatial features were seriously limited, the workflow appeared ‘chaotic’. Other resources that influenced the distribution of work included the number of staff available for dispensing, support staff such as counter assistants or drivers, and dispensing terminals. Some sites had more human resources than technical resources. This caused jostling between staff when a technical resource was required.

In organisation and prioritisation of work, the pharmacies organised work by setting out operating protocols that facilitated the distribution of resources and fluidity in the workflow. Whilst some had standardised protocols such as using colour-baskets to prioritise work, others customised protocols depending on which resources were available. As a result, dispensing journeys of prescriptions varied widely between sites. Time taken to dispense each prescription (usually walk-in prescriptions) was important concerning any interruptive process not directly related to this task. Interruptions (caused by telephone calls, urgent walk-in customer queries, lack of available computer terminals, and social interactions between staff and/or customers) all contributed to the time taken to dispense medicines.

Occupational tasks showed how autonomy, social interactions and multitasking influenced the dispensing process. There was a high level of multi-tasking in environments where one person was in charge from order entry to dispensing. In other environments, job sharing was high as it took up to four people to dispense. In some pharmacies, social activities were allowed during dispensing such as personal telephone calls, conversations between staff about non-dispensing issues and other non-work-related activities such as going on the Internet and to check personal emails.

In relation to engagement with technical systems of work, how staff used, and engaged with the technical system of work, influenced how work was propelled and practiced. In some pharmacies, technical systems such as using colour-baskets or assigning staff to specific dispensing terminals or even (in one pharmacy) using a robot to dispense, played a major part in how work was organised and approached. Other pharmacies integrated as little as possible with technologies.

This means that although the common goal of all the sites was to dispense safely, different adaptations of social and technical elements meant unique appropriation of operating procedures in the socio-technical context. Therefore, while all the pharmacies used social and technical elements in dispensing, some pharmacies used more social elements than technical elements and vice versa.

### Socio-technical approaches to work: developing a model of work practice

To conceptualise the socio-technical organisation of pharmacies, we analysed how each pharmacy used its own social and technical elements in each theme. For example, in each case study, we considered the following: how were the technical tools used to prioritise or organise work?; were there fewer staff compared to the number of services offered by the pharmacy and therefore high multi-tasking in the dispensing journey?; how much autonomy did the pharmacist have to influence work practice; did the staff socialise (by chatter) during dispensing?; did everyone have a designated task; were there technical protocols such as using coloured baskets, and were they adhered to?; did the staff share computers or work stations?; were there support staff such as designated counter assistants or delivery drivers?, and how was the space organised? The findings were mapped onto a socio-technical spectrum that ranged from highly social at one end of the spectrum to highly technical at the other end of the spectrum. In this phase, the focus was on how individual pharmacies repetitively and collectively used their own socio-technical elements by comparing them on the spectrum.

**Figure 3:**
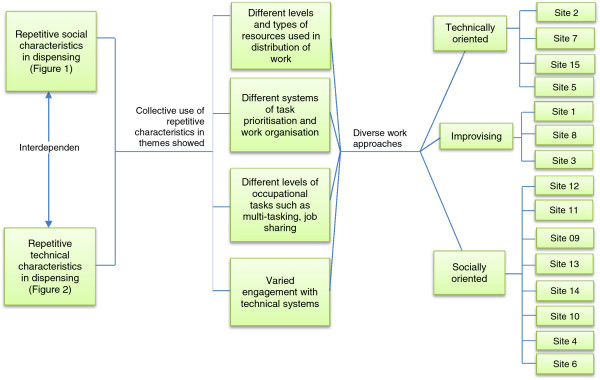
Socio-technical model of pharmacies studied.

As a result, how each pharmacy used their own socio-technical elements for dispensing showed a cluster of different approaches pharmacies used. For example, as pharmacy number two used eight computers, two robotic terminals, and a robot, its position on the spectrum regarding *engagement with technical systems* was ‘very technical’. The same pharmacy used standardised dispensing protocols such as using strict colour-basket system to prioritise work, staff multi-tasked less as they had designated tasks and individual dispensing stations. They used the technical elements to manage the workflow. Hence the work practice of this pharmacy, in both repetitive and collective use of socio-technical elements for dispensing, clustered at the technical end of the spectrum. The pharmacies that relied more on the people element in all the themes clustered at the social side of the spectrum. Some pharmacies however did not show a direction and were mostly clustered towards the middle of the spectrum. Through mapping, the 15 pharmacies showed three common approaches to dispensing. We have called these the technically oriented approach, the socially oriented approach, and the improvising approach. Four pharmacies were technically oriented, three were improvising and seven were socially oriented (Figure 3).

In the technically oriented approach, dispensing depended more on the technical than the social elements. The technical elements that were used to propel work included ‘hi-tech artefacts’ such as a robot (in one of the pharmacies), advanced pharmacy manager software, advanced problem-solving software, system remote problem-solving tools and system calibrating support. These were backed by ‘hi-tech work protocols’ such as prioritising work through dispensing baskets, standardised communication systems between staff, and between staff and customers, regimented transport system for prescription collection and delivery, highly organised medicines storage equipment and organised physical space. The human resource available for the workload was strongly aided by the technical elements. Whilst there were elements of social activity, these were kept to a minimum and were usually work-related social interaction such as tutoring pharmacy students, advising staff and customers on work issues, and briefing proprietors or external sources such as general practices and wholesale drug companies. Work appeared to halt if a technical element broke down. For example, the following extract made in a field note at a site that fell into this category. “*The high level of electronic engagement is seen by the pharmacist as advantageous to the pharmacy’s work processes and therefore is heavily relied upon…. Without the electronic facility, the dispensing process from the staff’s perspective becomes laboured*” (Case 07). The pre-registration (Pre-Reg) pharmacist from Case 07 confirmed this observation in the follow up interview from this example: “…*like this morning, our computer crashed. It wasted like ten, fifteen minutes of our time. In that time—obviously if the computer crashes you can’t dispense, either”* (*Case 07–02 Pre-Reg*).

In the socially oriented approach, the social elements mattered more than the technical elements. Staff relied on creating their unique social structure within which they could interact in order to propel work. Operational interaction between the hierarchies of human resource was central to effectively achieving dispensing. Frequent (and sometimes personal) interactions occurred between people delivering prescriptions (customers and delivery drivers), and pharmacy staff (counter assistants, dispensers, pharmacist and sometimes pharmacy proprietors). Some technical elements were used to enhance or strengthen the social structure such as bonding by discussing television programmes watched at home, or listening to music whilst carrying out work. This approach seems to focus on staff being ‘there’ for each other and the customers as one happy family through their unique social structure. The tight network of people made allowances for each other and was used to propel work. Members of staff appeared to have indifferent attitudes towards technologies and viewed them as just tools to contend with which was not integral to their work practice. For example, in relation to using colour-baskets to prioritise work, it was openly admitted in sites that fell into this category that the system was not strictly adhered to. The potential to adopt EPS2 did not excite attitudes as is shown by two interview extracts from dispensers in this category. *“I don’t really know what it entails. All it means to me is that instead of me going out and picking up [prescriptions from surgeries], it means I can be here to help do other things.”(Case 08–04, Dispenser); and, “I don’t know what it’s about really. It’s supposed to be people and technology (LAUGHS)..I don’t really know. Just saving paper, I guess. Other than that I don’t really see the benefits of it.. I think obviously it will help the customers. It will save them time.” (Case 06–02, Dispenser).*

In the improvising approach, dispensing can come across to the untrained eye as chaotic as it does not appear to have a particular approach or organisation to work; and uses every resource available to aid work in an apparently unsystematic way. The sites that took this type of approach were pharmacies that were, with limited resources, trying to achieve high work output such as offering a ‘collection and delivery’ service to tight deadlines, large-scale repeat dispensing, and medicines use review besides dealing with walk-in customers. This approach exploited any artefact available in the environment to carry out work by using technical aspects such as ‘hi-tech’ software and hardware, and social elements such as multi-tasking; but with staff trying to simultaneously control several processes. Organisation of work appeared unstructured and dispensing was often interrupted several times during the dispensing process as staff left current work to attend other work. The interruptions were more frequent in pharmacies that fell into this category than other approaches. Extract from field note on Case 03 demonstrates an example (FP10 is the prescription and CA the Counter Assistant):

*“A walk in-comes in with an FP10 Form. Counter Assistant/dispenser collects form and picks up medicines from the shelf. The medicines are taken to pharmacist who checks it and oks it. The labels are printed by pharmacist who puts the medicines into a basket. While this is going on, a customer walks in with a blister problem and asks for advice. The dispenser asks for an advice from pharmacist. They both stop what they were doing to advise customer. Pharmacist then comes back to finish dispensing. By this time a driver had delivered some medicines and needed a signature. Pharmacist goes and signs it and is distracted by another task. The dispenser is still seeing to the lady with blisters. Pharmacist then comes to label the medicines before packaging for dispensing. It is now 10.01*”.

Disruption to workflow appeared caused by insufficient resources available to match the many services provided which pressurised the socio-technical elements. Consequently, this appeared to impact negatively on every work activity as delays snowballed into other activities and affected the workflow. In pharmacies that fell into this category, staff hoped that the EPS2 will be the answer to their problems as this interview extract demonstrates: *“I just think it might be able to regulate our timings through the day so we can actually not be pressurised when we get all the prescriptions down at once from the doctor’s surgery, which happens at the moment. We can get like, over 100 items can come down at once that we have to dispense. Whereas this way, electronically, I’m hoping that as they come down like overnight, then we’ve got all morning to get them dispensed, whereas at the moment when they come down say, 12 o’clock I’ve only got an hour to get them done and get the deliveries done.” (Case 03–01, Pharmacist).*

Table 2 summarises key features of the three approaches from the socio-technical spectrum.

**Table 2:** Socio-technical model of community pharmacy work approaches

**Themes**	**Socially oriented**	**Improvising**	**Technically oriented**
*Resources*			
**Spatial resource** [1. Physical space; 2. Storage; 3. Assembling medicines space]	Adequate in two spatial resources or less	Adequate in two spatial resources or less	Adequate in two spatial resources or more
**Support staff** [ e.g. CA, collection & delivery drivers]	Dispenser is also a member of support staff	Dispenser is also a member of support staff	Designated support staff
**Number of dispensing staff in relation to dispensing duties**	One or multiple member of staff allocated to one dispensing duty at a time (e.g. walk-in)	One member of staff allocated up to three dispensing duties at a time (e.g. walk-in, cassettes, delivery prescriptions)	One member of staff allocated to one dispensing duty at a time (e.g. walk-in)
*Organisation of work*			
**Workload** [Observed activities staff simultaneously engaged in at peak periods]	Walk-ins, Low levels of repeat prescription processing	Walk-ins^b^, MURs, cassette fillings, batch repeat prescriptions (same day delivery), shelve restocking	Walk-ins, batch repeat prescriptions, cassette fillings, shelve restocking
**Prioritisation** [Simultaneous dispensing work undertaken by staff]	1 activity prioritised at a time	3 or more activities undertaken simultaneously by one staff	3 or more activities undertaken simultaneously by different staff
**Dispensing procedures**	Customised protocol	Unstructured protocol	Regimented Protocol
	*Work adapted to skills of people present*	*Protocols present but not adhered to*	*Work breaks down if protocol not strictly followed*
**Non dispensing activities**	Moderate-High	High	Low–Moderate
**Dispensing journey in minutes**^**c**^	3–8	10–14	2–5
*Occupational*			
**Multi-tasking**	Medium	High	Low–Medium
**Number of items dispensed**	Low–Moderate	Moderate-High	Moderate-High
	*Up to 150 per day*	*Over 150 per day*	*0ver 150 per day*
**Pharmacist autonomy**	Medium-low	High	Medium–High
*Technology*			
**Technical support**	Low	Medium to High	High
**Key technological systems**	Pharmacy Manager System (PMS)^d^	PMS	PMS
	AND/OR	AND one of the following	AND one or more of following
	Colour-basket system	Colour-basket system, Computerised Prescriber Order Entry (CPOE)	CPOE, Robot, Central computerised prescription managing hub (chain)
			AND/OR
			Colour-basket system
**Examples of supporting technologies**	Extra computer	Extra computer, colour-labels	Extra computer, remote technical support, technical calibrators, colour-labels
*Workflow*			
**Workflow**	Adaptive	Unpredictable	Methodical
	**Depends more on social elements**		**Depends more on technical elements**

^b^ Walk-ins contain acute prescriptions and repeat prescriptions delivered by the customer.

^c^ We use ‘dispensing journey’ to record when prescription is accepted by the pharmacy to when it is dispensed to the customer.

^d^ Software and associated hardware (e.g. label printer) used to manage dispensing.

### Socio-technical organisation as a factor in EPS2 implementation

We found that community pharmacies’ adapted their operating procedures for dispensing in various intricate ways depending on the socio-technical approach used in work practice. The Electronic Prescription Service Release 2 is a technical element that will be adopted into the core of dispensing in terms of downloading electronic prescriptions that are stored on national database called the Spine, and dispensing and endorsing the prescriptions through automated means [1]. The EPS2, through automation, has the potential to streamline workload and increase fluidity of workflows for pharmacies as key functions [3]. As community pharmacies prepare to adopt the EPS2, we discuss ways in which the socio-technical organisation of pharmacies could become a factor in a successful adoption.

The technically oriented approach exploited technical systems of work fully, and seemed ready for any future technological additions such as the EPS2, as it would be exploited fully to benefit work. According to Hakken [29] and Westerling et al. [30], computers are used in the centrality of work as a strategy to economic revitalisation. Pharmacies that took a technically oriented approach to work offered many dispensing services and dispensed many items therefore could be construed as economically driven. With EPS2, these pharmacies may be able to enhance and speed up processes, especially through advance repeat dispensing, to attain even greater heights of work. In addition, the techno-innovative outlook of these pharmacies would be enhanced. However, EPS2 could be one too many technical systems as the reliance on technologies could impose major challenges such as interference in workflow if the technical elements do not synchronise correctly with EPS2.

In a recent investigation on the strategic and long-term role of IT systems in community pharmacies, some experts asserted that the presence of IT is not a central value, but an essential tool for pharmacy service provision [30]. In the socially oriented approach, this view was evident as staff regarded technical systems as resources that aided work rather than being central to it. Introducing a technical element such as the EPS2 to the core of this work approach could dramatically influence how work is conducted, as a technical system would be brought into the centre of activities. Although pharmacies that currently use this approach manage dispensing service effectively, work could be improved by using the technical system to aid the human processes, thereby retaining the social structure on which it thrives. For example, processes could be speeded up with EPS2. However, such technical change could be too dramatic for staff and could result in rejection of the system as evidence shows how barriers such as user frustration and resistance are hindering Computerised Prescriber Oder Entry (CPOE) system implementation in United States of America [7].

In the improvising approach, how work was organised seemed the barrier to achieving tasks effectively, and negatively influenced every dispensing process. Whilst technologies were engaged with to achieve work, the unrealistic targets caused staff to improvise with any tool available. Introducing EPS2 has the potential to improve how work is organised in this approach. For example at the start of a day, pharmacies could download most of the day’s EPS2 prescriptions and start dispensing them before general practices open, allowing dispensing to be carried out more smoothly through the day, rather than peaking in GP hours. However, to take advantage of EPS2 benefits, these pharmacies will need to establish a coherent protocol that makes better use of both social and technological elements to stop the new system from being another obstacle in the unpredictable workflow.

The Department of Health in England has been increasing the clinical roles that it wishes pharmacists to undertake, and these will require more time spent in consultation rooms with patients. The introduction of EPS2 may allow these roles to be more easily undertaken. We note that while the model conceptualises work practice, daily work in community pharmacies encompass all these three approaches in subtle ways, although the small differences are what matter [31].

### Strengths

This qualitative study conceptualises pharmacy work practices in the social-technical context. The study employed an in-depth and innovative socio-scientific method and complemented it with rigorous multi-dimensional and multi-disciplinary validation techniques to ground the study. It offers a socio-scientific work practice model development that can be replicated and improved on by other studies beyond pharmacy practice discipline. It adds new knowledge to health services research, and pharmacy practice literatures in terms of methodology and findings.

### Limitations

Since the study employs ethnographical methods, findings could be construed as subjective. We acknowledge that the conceptual model offered could be further improved by using other studies to complement these findings, and studying more pharmacies in future research. While we focused on socio-technical elements of dispensing, this is not to say that other factors are not involved in pharmacy work approaches. Pharmacies were not observed throughout the full range of opening hours and days, and some activities may have happened outside the observed times.

## Conclusions

Our conceptual model shows that different approaches to work exist in community pharmacy work practice based on how various social, technical (and other elements) of the pharmacy are used to achieve work. Some made more use of the social elements; others made more use of the technical artefacts. From our ethnographic study, we empirically inform the debate on how EPS2 could ‘impact’ pharmacy work practice. Currently, NHS Connecting for Health plans to rollout EPS2 to all community pharmacies in England. When this happens, technically oriented pharmacies seem ready for integrating another technological service into the core of their work, although their total reliance on technical systems could expose them to major challenges such as interference in workflow if their technical elements do not work in synchronicity with EPS2. Improvising approach pharmacies have the potential to improve how work is organised through streamlining however, there is danger that the EPS2 could become another obstacle in the already unpredictable workflow. Socially oriented approach pharmacies could use the new technical element to aid the human processes although they might experience dramatic changes, as they would be forced to integrate a technical artefact into the core of their work practice.

## Abbreviations

EPS2: Electronic Prescription Service release 2; MUR: Medicines Use Review; NHS CfH: National Health Service Connecting for Health; Pre-Reg: Pre-registration Pharmacist.

## Competing interests

The authors declare that they have no competing interests.

## Authors’ contributions

JH facilitated the refinement of the research design, was the primary data collector of the study and collected data from all the 15 pharmacies. JH conducted the analysis and interpretation of the data together with AA and NB and other members of the team; and the lead writing of this paper.

AA co-authored and edited all the cases studies from the study, extensively contributed to the analysis and the interpretation and of the data and provided commentary on the writing up process. JW helped to design the study and gave constructive feedback on the initial stages of the manuscript preparation. NB contributed to the analysis and critical interpretation of the data analysis and provided key insights to improve the paper. All authors read and approved the final manuscript.

## Authors’ information

JH is a social scientist who specialises in Social Informatics – ethnological study of people’s (dis)engagement with technologies. She has a multi-disciplinary background in human geography, information science and information technology, and currently works in the Department of Social Science, Loughborough University. JH also holds an honorary lectureship with the Division of Primary Care, School of Community Health Sciences at the University of Nottingham.

AA is professor of Primary Health Care at the Division of Primary Care, Queens Medical Centre, University of Nottingham. Among other interests, he specialises in patient safety and the use of information technology to aid clinical practice, and has an extensive portfolio and high starred academic papers for both quantitative and qualitative research in this field. AA is also an active general practitioner in the city of Nottingham.

JW is a professor and a sociologist at Nottingham University Business School, The University of Nottingham. His research centres on the changing organisation and management of professional work, especially healthcare professions, in the context of contemporary public service reforms.

NB is professor of the Practice of Pharmacy at the Department of Practice and Policy, University College London School of Pharmacy, University of London. NB gave a critical insight throughout the research write up and critically edited this paper. NB is also a visiting professor of medication safety at Harvard Medical School and is a practising pharmacist.

## Pre-publication history

The pre-publication history for this paper can be accessed here:

http://www.biomedcentral.com/1472-6963/12/471/prepub

## Supplementary Material

Additional file 1 **Appendix 1.** Sample field notes to show observation format (Edited. Commercially sensitive and identifiable information removed). Click here for file
