# Supplementary material for: The socio-technical organisation of community pharmacies as a factor in the Electronic Prescription Service Release Two implementation: a qualitative study
Source: BMC Health Serv Res. 2012 Dec 20;12:471. doi: 10.1186/1472-6963-12-471 (PMC3551639; doi:10.1186/1472-6963-12-471)
Supplement: Additional file 1 — Appendix 1. Sample field notes to show observation format (Edited. Commercially sensitive and identifiable information removed). [file 1472-6963-12-471-S1.docx]

Appendix 1: Sample field notes to show observation format (Edited. Commercially sensitive and identifiable information removed).

Field notes* example – Site 03 (showing field notes for the timeline 9.30-11.00).

**Descriptive information**

| **Researcher(s**): | JH; assisted by RH on day two to record walk-in dispensing timing. |
| --- | --- |
| **Name and address of pharmacy** | - (Information removed) |
| **Type of pharmacy system**: | - (information removed) |
| **Number of staff present:** | Five at the start of observation |
| **Date (s) of observation** | • Feb 2010 (day removed for anonymity)  • Feb 2010 |
| **Number of Staff** | 5 |
| **Type of staff:** | One pharmacist, one pre-registration student, one pharmacy technician, one dispenser and one medicine counter assistant/dispenser. |
| **Computer/dispensing stations on site**: | Two dispensing stations |
| **Number of prescriptions dispensed/month** | - (information removed) |
| **Repeat% :Acute %** | 80%:20% |
| **Other technologies** | 2 printer; 2x label printers; telephones, CCTV camera, controlled drugs fridge, colour baskets |
|  |  |

**Floor layout**

(Identifiable information removed)

**Key dispensing activities**

- Medication Use Reviews
- Pick and collection prescriptions from nearby surgeries
- Repeat prescriptions
- Cassettes and batched orders
- Stock and product cataloguing

**Format of Prescription dispensing journey for ‘walk-in’ customers and ‘repeat prescription’ customers**

(Commercially sensitive information removed)

**Day 1: Showing observation from 9.25 to 11.00 as an example of field notes collected.**

**Note**: Medicines are all over the shelves and sink area. Community cassettes too

9.30 So far, a majority of the prescriptions dispensed are walk-ins with people picking up dispensed medicines (call backs).

9.47 A walk in-comes in with an FP10 Form. Counter Assistant/dispenser collects form and initials it, and picks up medicines from the shelf. The medicines are taken to pharmacist who checks it and oks it. The labels are printed by pharmacist who puts the medicines into a basket.

- While this is going on, a customer walks in with a blister problem and asks for advice. The dispenser asks for an advice from pharmacist. They both stop what they were doing to advise customer. Pharmacist then comes back to finish dispensing. By this time a driver had delivered some medicines and needed a signature. Pharmacist goes and signs it and is distracted by another task. The dispenser is still seeing to the lady with blisters. Pharmacist then comes to label the medicines (on the bottle) before packaging for dispensing. It is now 10.01.

-**Note**: So far, a majority of customers appear middle-aged and of both genders. They do not appear to mind the time taken to dispense. Some drop off the prescription, go out to shop and come back to pick up.

10.02 Pharmacist takes a customer to the consulting room for MUR (Medicines Use Review). She comes out to print some information and goes back to consulting room. She is asked a question by dispenser.

10.05 The walk-in customer from 9.47 is back to collect. Dispenser takes medicines and informs pharmacist who is still in the consulting room and dispenses medicine.

10.07 Customer comes in to pick-up prescription. He seemed well known to the dispenser.

10.08 A walk-in arrive customer arrives and queues. Counter assistant picks and initials prescription and gives it to the pre-reg student. Student picks medicine and put in basket for pharmacist who is now coming out from consulting room. Pharmacist advises student. Pharmacist start processing the prescription, packages the medicines goes over to the customer and has a little chat before dispensing at 10.10.

There are two walk-in customers at 10.11. They elect to call back for their medications in few minutes. They are advised it will take 10-15 minutes to prepare medicines.

**-Note**: - So far, staff seems familiar with most of the customers. It is a like a– social hub. Pharmacist seems to perform regular consultation duties with customers. It is 10.14 and there have been two consultations. Pharmacist prints their prescription records before consulting. Consultations so far take above five minutes. (**Added after day 1 observation**) Pharmacist revealed that a target of xxx MURs are required per year. When targets are met, there is pay rise for all the staff.

**Note**:- There is very little free space for processing medicines. Medicines are placed directly into baskets and baskets are piled on top of each other as there is very little space.

10.15 a dispensed medicine is picked-up by customer who dropped off prescription earlier.

10.20 phone rings and pre-reg student takes call. It was a customer asking if a particular medicine is ready for collection. He conveys message to pharmacist. Pharmacist promises to order medicine –

10.24 2^nd^ computer is off. Only one computer is being used for dispensing. Three elderly customers are in the shop front chatting. They are waiting for their prescriptions to be dispensed.

10.30 a customer walks-in with prescription. Pharmacist takes order and processes it. Customer waits and chats.

10.30 a new customer walks in and is greeted by name.

**Note**: so far customers are usually addressed by their first names.

10.35 Earlier walk-in customer from 10.11 comes to pick-up medication.

10.45 Customer comes in to pick up medication. Wrong pharmacy. Counter Assistant directs him to the right one.

**Note**: there are now fewer customers. CA and dispenser are doing date checking, and re-stocking shelves with medicines delivered by the drug delivery driver earlier.

10.55 2^nd^ computer is still off.

11.00 Customer comes in to buy medicine off-the-shelf. He is known the staff. Pharmacist asks him to come back later for MUR.
